# Associations between childhood body size and seventeen adverse outcomes: analysis of 65,057 European women

**DOI:** 10.1038/s41598-017-17258-5

**Published:** 2017-12-05

**Authors:** Jingmei Li, Mikael Eriksson, Wei He, Per Hall, Kamila Czene

**Affiliations:** 1 Genome Institute of Singapore, 60 Biopolis Street, Genome, #02–01, Singapore, 138672 Singapore; 2 Department of Surgery, Yong Loo Lin School of Medicine, National University of Singapore, Singapore, 119228 Singapore; 3 Karolinska Institutet, Department of Medical Epidemiology and Biostatistics, Box 281, 171 77 Stockholm, Sweden; 4 Department of Oncology, Södersjukhuset, 118 84 Stockholm, Sweden

## Abstract

Large childhood body size has been consistently shown to be associated with decreased breast cancer risk. However, it is important to consider the effects of a large childhood body size on other adult diseases. It is not clear if the associations between childhood body size and adult diseases will persist if they later attain healthy weight. The associations between body size at age 7 and 17 adverse outcomes in adulthood were examined using Cox models in a Swedish study of 65,057 women. Large body size at age 7, when compared to small body size, was associated with decreased risk for breast cancer (HR [95% CI]: 0.81 [0.70–0.93]) and increased risks for anorexia (2.13 [1.63–2.77]) and bulimia (1.91 [1.35–2.70]). Neither adjusting for adult BMI nor restricting the dataset to lean adults (BMI < 25 kg/m^2^) attenuated the associations. While large body size at age 7 by itself was positively associated with increased risks of diabetes (1.34 [1.16–1.55]), PCOS (1.69 [1.13–2.51]) and hypertension (before age 60), the associations were no longer significant after controlling for adult BMI. No clear associations were found with the remaining adverse outcomes (cervical, uterine, melanoma, colon cancer, depression, ovarian cyst, stroke, hyperlipidemia, heart failure, myocardial infarction, and angina pectoris).

## Introduction

Obesity is classified as a chronic disease by the World Health Organization (WHO) (2000 report “Obesity: Preventing and Managing the Global Epidemic”), described as being prevalent in both developed and developing countries, and affecting children as well as adults. Various measures of both adult and childhood anthropometry such as BMI, height, waist-to-hip ratio, and body size have been extensively studied as risk factors for many adult diseases. In particular, research on the effect of childhood obesity on clinical disease in adulthood is of public health interest, as the potential for early intervention is high.

Childhood body size, in particular, is an interesting early life risk factor, as it has been consistently shown to confer protection against both pre- and postmenopausal breast cancer^1,2^. This counterintuitive link has caught media attention and sparked public interest. However, larger children are at greater risk of being overweight or obese when they get older, which may in turn confer a greater risk of poor health due to other causes and premature death in adulthood^3,4^. Diabetes, stroke, hypertension, angina and heart diseases which are commonly associated with adult obesity have also been demonstrated to be significantly associated with childhood body size^5–13^. In addition, potential links have been found between large childhood body size and increased risks of several cancers, which includes malignancies in the endometrium, colon, thyroid, liver, brain, and spine^14–20^. Childhood stature has also been associated with higher predisposition to adult melanoma^21^.

While it is unclear if estimates of simple body size are better indicators of adiposity than BMI, the use of somatotypes to measure childhood anthropometry has several advantages over childhood BMI. For example, BMI is generally defined in adults as an index of adiposity that is largely independent of stature; but BMI in children is highly influenced by height^22^. In addition, BMI is not only correlated with total body fat and percent body fat^23–26^, but also with fat-free mass^22^. Childhood BMI has been shown to perform poorly to moderately well in identifying children with excess adiposity, thus missing a large proportion of those who are truly overweight, as determined from percent body fat^27–29^. Women may also find it easier to recall approximate body size during childhood than BMI.

Although childhood body size is recognized as an early-life risk factor for many adult diseases, most studies to date have presented the associations with different diseases separately in different reports. However, most of these articles do not emphasize enough that associations is not causation, and that while large body size during childhood may decrease the risk of certain conditions such as breast cancer, it could also elevate the risks of other negative health outcomes. With the rise of large cohort studies, it is timely to examine the effect of childhood body size on multiple adult health conditions in the same study to give a well-rounded overview of the risk factor. We thus examined a wide range of long-term consequences of a large body size during childhood on later life well-being in a large Swedish study comprising 65,057 women. The main aim of this study was to clarify the association between childhood body size and different diseases using the same set of women. We also looked at whether childhood body size is an independent risk factor after adult BMI is taken into account. As it is unclear if the associations between childhood body size and adult diseases will persist if the children later attain healthy weight, we performed subset analyses in women who were not overweight (<25 kg/m_2_) in adult life and also in women who were neither overweight nor of a large body size during adolescence (age 18).

## Methods

### Study population

**Figure 1:**
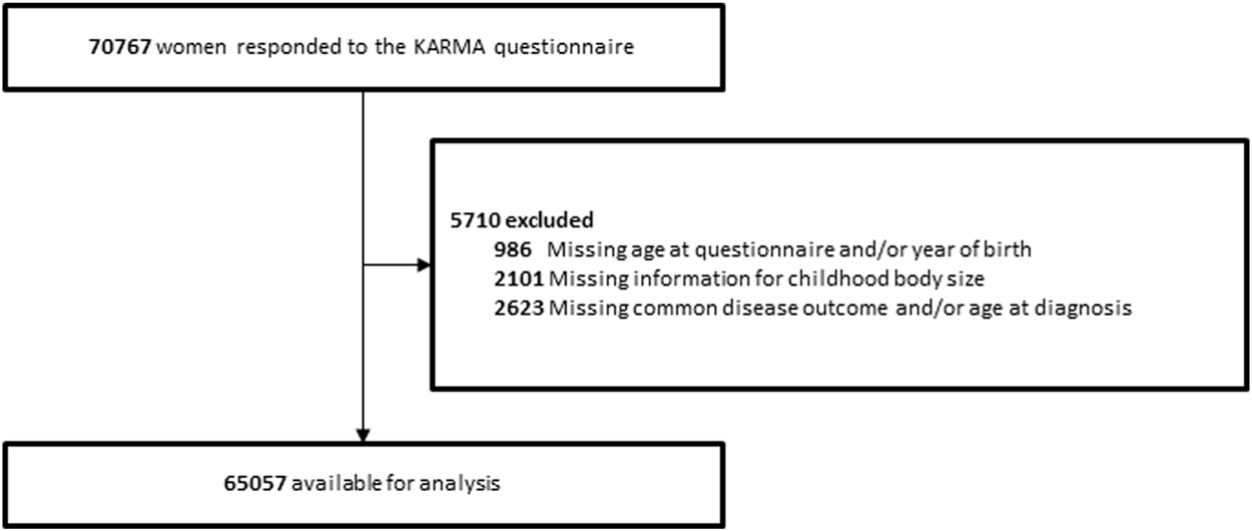
Flow diagram of how the analytical cohort was derived.

The KARMA (KARolinska MAmmography Project for Risk Prediction of Breast Cancer, http://karmastudy.org/) study is a well-characterized breast cancer cohort study in Sweden^30^. All women who underwent screening or clinical mammography between January 2011 and March 2013 at four hospitals (Stockholm South General Hospital, Helsingborg Hospital, Skåne University Hospital and Landskrona Hospital) were invited to participate. The mean ages of women at time of invitation and recruitment for the parent cohort were 53.7 (standard deviation [SD] 9.9) and 54.6 (SD 10.0) years, respectively. Approximately 53% of the recruited women were postmenopausal. Each participant gave informed consent and this study was approved by the ethical review board at Karolinska Institutet. A flow diagram of how the analytical cohort of 65,057 women was derived after the exclusion of 5,710 women with missing information on age, childhood body size and common diseases is shown in Fig. 1.

### Assessment of childhood body size

Body size at ages 7 and 18 were assessed using the Stunkard Figure Rating Scale^31^ in the KARMA questionnaire. The well-validated nine-level pictogram illustrates body sizes ranging from extreme thinness (category 1) to obesity (category 9)^32,33^, which we grouped into three major categories (small [categories 1 and 2], medium [categories 3 and 4], large [categories 5 and above])^34^ based on visual assessments of how similar the body sizes were. The major categories of childhood body size (small, medium and large) were treated as a semi-continuous variable (coded 0, 1, and 2) to estimate linear trend. Childhood body size was also evaluated as a continuous variable (nine levels). Childhood BMI was not collected.

### Linkage to Swedish Cancer Register

Linkage to the Swedish Cancer Register (last updated 2013–12–31) was performed using Personal Identity Numbers, a ten–digit number that is unique for every resident in Sweden and used in all health-based registers^35^. Established in 1958, the Swedish Cancer Register is known for its high quality and coverage^36^. Although the Swedish Cancer Register includes all cancers (*n* = 5,346 in our study), we included only five specific sites (breast [*n* = 2,733], cervix uteri [*n* = 222], corpus uteri [*n* = 259], melanoma [*n* = 551] and colon [*n* = 235]) in our analyses in view of the number of events available for meaningful analyses.

### Self-reported adverse outcomes derived from questionnaire

Participants were asked “Have you ever been diagnosed with any of the following by a medical doctor?” for 13 non-cancer adverse outcomes: diabetes, hypertension, hyperlipidemia, myocardial infarction, angina pectoris, heart failure, stroke, pre-eclampsia, polycystic ovary syndrome, ovarian cysts, depression, bulimia and anorexia. Corresponding years of diagnosis were also self-reported. Pre-clampsia was not included as an outcome of interest as it is a condition that occurs only during pregnancy.

### Statistical analysis

Using Cox proportional hazards regression models, the associations between childhood body size (small, medium and large) and 17 adverse outcomes (5 cancers and 12 common adult diseases) were assessed. As the risk factors for pre- and postmenopausal breast cancer may be different^37^, we performed stratification by menopausal status. Chronological age was chosen as the underlying time scale. All analyses were stratified by year of birth (≤1950, 1951–1960, 1961 and later) to control for cohort effects. Women enter the analysis at age 7 (left-truncation). For each outcome trait studied, follow-up time was until age at diagnosis of the trait or age at questionnaire. The proportionality assumption of Cox regression was verified using scaled Schoenfeld residuals (cox.zph). The assumption of proportionality was violated for one outcome – hypertension. The time-dependent hazard ratios for hypertension were therefore estimated using flexible parametric survival models (stpm2 module in STATA).

To investigate whether the associations between childhood body size and common diseases in adulthood were independent of adult BMI, we adjusted for adult BMI. To investigate whether childhood body size was associated with common diseases in adulthood for women who were not overweight in adult life, we repeated the analyses in a subset of women with adult BMI < 25 kg/m^2^. To investigate whether childhood body size was associated with common diseases in adulthood for women who were neither overweight in adult life nor of a large body size during adolescence, we further repeated the analyses in a subset of women with adult BMI < 25 kg/m^2^ and of either a small or medium body size at age 18. In summary, we ran four models for each adverse outcome: no adjustment (*), adjusted for BMI at questionnaire (continuous, kg/m^2^) (†), no adjustment for a subset of women with BMI less than 25 kg/m^2^ at questionnaire (‡) and no adjustment for a subset of women with adult BMI less than 25 kg/m^2^ and either small or medium body size at age 18 (§). The described analyses were performed in R (version 3.3.1)^38^ unless otherwise stated. All analyses were performed in accordance with relevant institutional and national guidelines and regulations.

### Restricting analysis to breast cancer controls

In Sweden, the proportion of breast cancer among all incident cancer cases (2010–2014) is 27.6%^39^. As women attending clinical mammography were also invited to participate in the KARMA study in addition to women attending screening, breast cancer cases are overrepresented in the KARMA study, contributing to over half of all cancer cases studied (Supplementary Table 1). In this study, the overlap between women with any common disease and breast cancer ranges from 5.5% to 9.6%. Given that childhood body size has been reported to be associated with breast cancer risk in opposite directions to those of other health outcomes, we repeated all analyses on a subset of 62,324 non-breast cancer participants.

## Results

### Participant characteristics

Table 1 describes participant characteristics of the 65,057 women included in the study. The average age at study entry was 55.1 years. One in five women had a diagnosis of hypertension, with the average age at diagnosis at 50.8 years. Other common health outcomes include depression (14.8%), hyperlipidemia (10.6%) and breast cancer (4.2%). Mean ages at diagnosis for diabetes, stroke, angina pectoris, myocardial infarction and heart failure were 48.9, 54.5, 55.2, 56.4 and 56.8 years, respectively. Health conditions with mean ages at diagnosis below 45 years include depression (41.5 years), ovarian cyst (36.1 years), PCOS (29.4 years), bulimia (25.3 years) and anorexia (19.6 years).

**Table 1:** Characteristics of study population (*n* = 65,057) from Karolinska Mammography Project for Risk Prediction of Breast Cancer (KARMA), recruited between 2011 and 2013.

Age at entry (years), mean (SD)	55.1 (10.0)	
Age at menarche (years), mean (SD)	13.1 (1.5)	
Irregular menstrual cycle during adult life, *n* (%)	7626 (11.3)	
Physical activity at age 18 (h/week), *n* (%)		
0	2749 (4.2)	
<1	7294 (11.2)	
1–2	17207 (26.4)	
3–5	17377 (26.7)	
5+	14570 (22.4)	
Education, *n* (%)		
Elementary	7518 (11.6)	
Intermediate	17795 (27.4)	
College	30012 (46.1)	
Other	9559 (14.7)	
Number of children, *n* (%)		
0	8304 (12.8)	
1	9622 (14.8)	
2	30996 (47.6)	
3+	16126 (24.8)	
Ever regular smoke, *n* (%)	34423 (52.9)	
Body mass index at questionnaire (BMI, kg/m^2^), mean (SD)	25.2 (4.2)	
Body size at age 7, *n* (%)		
1	15074 (23.2)	
2	20032 (30.8)	
3	14091 (21.7)	
4	9433 (14.5)	
5	4592 (7.1)	
6	1511 (2.3)	
7	260 (0.4)	
8	53 (0.1)	
9	11 (<0.1)	
Cancer outcomes, *n* (%)		Average age at diagnosis (years), mean (SD)
Breast	2733 (4.2)	55.4 (10.1)
Cervical cancer	222 (0.3)	41.9 (10.9)
Uterine	259 (0.4)	58.8 (7.5)
Melanoma	551 (0.8)	47.8 (13.1)
Colon	235 (0.4)	55.0 (14.3)
Non-cancer outcomes, *n* (%)		
Depression	9642 (14.8)	41.5 (11.4)
Anorexia	498 (0.8)	19.6 (7.1)
Bulimia	318 (0.5)	25.3 (9.7)
PCOS	217 (0.3)	29.4 (6.8)
Ovarian cyst	4325 (6.6)	36.1 (12.7)
Diabetes	1805 (2.8)	48.9 (15.5)
Hypertension	12695 (19.5)	50.8 (11.7)
Hyperlipidemia	6869 (10.6)	54.3 (10.1)
Stroke	689 (1.1)	54.5 (11.8)
Heart failure	369 (0.6)	56.8 (13.0)
Myocardial infarction	479 (0.7)	56.4 (9.4)
Angina pectoris	570 (0.9)	55.2 (10.7)

Table 2 shows the associations between childhood body size and five common cancers in adulthood (breast, cervical, uterine, skin and colon). A significant dose-dependent inverse relationship was observed between childhood body size and breast cancer. Compared to small childhood body size, large childhood body size was associated with a 19% decrease in breast cancer risk in the unadjusted model (HR_large vs small_*: 0.81 [0.70 to 0.93], P_continuous_* = 0.005). This association was found to be independent of adult BMI (HR_large vs small_†: 0.79 [0.68 to 0.91], P_continuous_† = 0.001). Neither restricting the dataset to adults who were not overweight (<25 kg/m^2^) (HR_large vs small_‡: 0.73 [0.59 to 0.92], P_continuous_‡ = 0.011) nor further restricting the dataset to adults who were not of a large body size at age 18 (HR_large vs small_ **§**: 0.65 [0.49 to 0.86], P_continuous_ **§** = 0.013) attenuated the association. The effect sizes of the associations between childhood body size and adult breast cancer risk stratified by menopausal status were appreciably different in the crude and BMI-adjusted analyses. However, the reduced breast cancer risk appeared to be more pronounced for postmenopausal breast cancer in a subset of women who were lean during adolescence and adulthood (HR_large vs smal_ **§**_l_: 0.55 [0.38 to 0.81], P_continuous_ **§** = 0.050). No clear association was observed between childhood body size and any of the other cancers studied (cervical, uterine, melanoma and colon).

**Table 2:** Hazard ratios (HR) and corresponding 95% confidence intervals (CI) for the associations between childhood body size and different cancers in 65,057 women.

Cancer Outcome	Body size	*n*	Crude	Adjusted for BMI	*n*‡	Subset, adult BMI < 25 kg/m^2^, i.e. lean during adulthood	*n*§	Subset, adult BMI < 25 kg/m^2^ and small/medium at 18, i.e. lean during adolescence and adulthood
			HR (95% CI)*	HR (95% CI)†		HR (95% CI)‡		HR (95% CI)§
Breast	Small	1573	1.00 (Reference)	1.00 (Reference)	862	1.00 (Reference)	852	1.00 (Reference)
	Medium	942	0.94 (0.87 to 1.02)	0.93 (0.86 to 1.01)	428	0.93 (0.83 to 1.05)	399	0.94 (0.84 to 1.06)
	Large	218	**0.81 (0.70 to 0.93)**	**0.79 (0.68 to 0.91)**	84	**0.73 (0.59 to 0.92)**	51	**0.65 (0.49 to 0.86)**
	Trend	2733	**0.92 (0.86 to 0.97)**	**0.90 (0.85 to 0.96)**	1374	**0.89 (0.82 to 0.97)**	1302	**0.88 (0.80 to 0.97)**
	Continuous	2733	**0.96 (0.93 to 0.99)**	**0.95 (0.93 to 0.98)**	1374	**0.95 (0.91 to 0.99)**	1302	**0.94 (0.90 to 0.99)**
Breast	Small	514	1.00 (Reference)	1.00 (Reference)	306	1.00 (Reference)	302	1.00 (Reference)
(Premenopausal)	Medium	325	0.95 (0.83 to 1.09)	0.95 (0.83 to 1.10)	154	0.94 (0.78 to 1.15)	143	0.96 (0.78 to 1.17)
	Large	80	0.84 (0.67 to 1.07)	0.84 (0.67 to 1.07)	35	0.89 (0.63 to 1.27)	23	0.84 (0.55 to 1.29)
	Trend	919	0.93 (0.84 to 1.03)	0.93 (0.84 to 1.03)	495	0.95 (0.82 to 1.09)	468	0.94 (0.80 to 1.10)
	Continuous	919	0.96 (0.91 to 1.00)	0.96 (0.91 to 1.00)	495	0.96 (0.89 to 1.03)	468	0.95 (0.88 to 1.03)
Breast	Small	1044	1.00 (Reference)	1.00 (Reference)	545	1.00 (Reference)	539	1.00 (Reference)
(Postmenopausal)	Medium	608	0.94 (0.85 to 1.04)	0.92 (0.83 to 1.02)	269	0.93 (0.80 to 1.08)	251	0.94 (0.81 to 1.10)
	Large	138	**0.80 (0.67 to 0.96)**	**0.78 (0.65 to 0.93)**	49	**0.66 (0.50 to 0.89)**	28	**0.55 (0.38 to 0.81)**
	Trend	1790	**0.91 (0.85 to 0.98)**	**0.90 (0.84 to 0.97)**	863	**0.87 (0.78 to 0.97)**	818	**0.85 (0.76 to 0.96)**
	Continuous	1790	0.97 (0.93 to 1.00)	**0.96 (0.92 to 0.99)**	863	**0.95 (0.90 to 1.00)**	818	0.94 (0.89 to 1.00)
Cervical	Small	130	1.00 (Reference)	1.00 (Reference)	78	1.00 (Reference)	78	1.00 (Reference)
	Medium	68	0.79 (0.59 to 1.06)	0.80 (0.59 to 1.07)	31	0.74 (0.49 to 1.12)	30	0.77 (0.51 to 1.17)
	Large	24	1.03 (0.66 to 1.58)	1.03 (0.66 to 1.60)	15	1.49 (0.85 to 2.58)	8	1.13 (0.55 to 2.35)
	Trend	222	0.93 (0.76 to 1.14)	0.93 (0.76 to 1.14)	124	1.04 (0.79 to 1.36)	116	0.91 (0.67 to 1.25)
	Continuous	222	0.96 (0.87 to 1.06)	0.96 (0.87 to 1.06)	124	1.00 (0.87 to 1.14)	116	0.93 (0.79 to 1.08)
Uterine	Small	151	1.00 (Reference)	1.00 (Reference)	70	1.00 (Reference)	67	1.00 (Reference)
	Medium	89	0.94 (0.72 to 1.22)	0.87 (0.67 to 1.13)	30	0.80 (0.52 to 1.22)	23	0.68 (0.42 to 1.09)
	Large	19	0.76 (0.47 to 1.22)	0.66 (0.41 to 1.06)	6	0.63 (0.27 to 1.44)	3	0.47 (0.15 to 1.49)
	Trend	259	0.90 (0.74 to 1.09)	0.83 (0.69 to 1.01)	106	0.79 (0.58 to 1.09)	93	0.68 (0.46 to 1.00)
	Continuous	259	0.97 (0.89 to 1.07)	0.94 (0.85 to 1.03)	106	0.93 (0.80 to 1.08)	93	0.86 (0.72 to 1.03)
Melanoma	Small	301	1.00 (Reference)	1.00 (Reference)	186	1.00 (Reference)	182	1.00 (Reference)
	Medium	184	0.94 (0.78 to 1.13)	0.95 (0.79 to 1.14)	93	0.93 (0.73 to 1.20)	84	0.93 (0.71 to 1.20)
	Large	66	1.25 (0.95 to 1.63)	1.26 (0.96 to 1.65)	24	0.99 (0.64 to 1.51)	18	1.08 (0.67 to 1.76)
	Trend	551	1.06 (0.93 to 1.20)	1.06 (0.94 to 1.21)	303	0.97 (0.81 to 1.16)	284	0.98 (0.81 to 1.19)
	Continuous	551	1.02 (0.96 to 1.09)	1.03 (0.96 to 1.09)	303	0.98 (0.90 to 1.07)	284	0.99 (0.90 to 1.09)
Colon	Small	125	1.00 (Reference)	1.00 (Reference)	67	1.00 (Reference)	66	1.00 (Reference)
	Medium	83	1.06 (0.80 to 1.39)	1.06 (0.80 to 1.40)	40	1.12 (0.76 to 1.66)	36	1.10 (0.74 to 1.66)
	Large	27	1.28 (0.85 to 1.94)	1.28 (0.84 to 1.95)	15	1.69 (0.97 to 2.97)	9	1.49 (0.74 to 2.98)
	Trend	235	1.11 (0.92 to 1.34)	1.11 (0.92 to 1.34)	122	1.24 (0.96 to 1.61)	111	1.17 (0.87 to 1.57)
	Continuous	235	1.05 (0.96 to 1.15)	1.05 (0.96 to 1.15)	122	1.12 (0.99 to 1.28)	111	1.11 (0.96 to 1.28)

Statistically significant associations (P < 0.05) are presented in bold. ^*^Stratified by year of birth (1950, 1951–1960, 1961 and later). ^†^Stratified by year of birth and adjusted for body mass index (BMI, kg/m^2^, continuous) at questionnaire. ^‡^Subset of women with adult BMI less than 25 kg/m^2^, stratified by year of birth. ^**§**^Subset of women with adult BMI less than 25 kg/m and either small or medium body size at age 18, stratified by year of birth.

Table 3 shows the associations between childhood body size and eleven common diseases (depression, anorexia, bulimia, PCOS, ovarian cyst, diabetes, hyperlipidemia, stroke, heart failure, myocardial infarction and angina pectoris). In the crude analyses, when compared to a small childhood body size, a large childhood body size was associated with nearly two-fold increased risks with both anorexia (HR_large vs small_*:2.13 [1.63 to 2.77], P_continuous_* < 0.001) and bulimia (HR_large vs small_*:1.91 [1.35 to 2.70], P_continuous_* < 0.001). Similarly, larger childhood body size was associated with increased risk of diabetes and PCOS in a dose-dependent manner (HR_large vs small_ *: 1.34 [1.16 to 1.55], P_continuous_ * = 0.021 and HR_large vs small_*: 1.69 [1.13 to 2.51], P_continuous_* = 0.007, respectively). The increased risks for diabetes and PCOS conferred by larger childhood body size were attenuated when adult BMI was taken into account (HR_large vs small_†: 0.97 [0.84 to 1.13], P_continuous_† = 0.005 and HR_large vs small_†: 1.25 [0.83 to 1.88], P_continuous_

**Table 3:** Hazard ratios (HR) and corresponding 95% confidence intervals (CI) for the associations between childhood body size and self-reported common diseases in 65,057 women.

Outcome	Body size	*n*	Crude	Adjusted for BMI	*n*‡	Subset, adult BMI < 25 kg/m^2^, i.e. lean during adulthood	*n*§	Subset, adult BMI < 25 kg/m^2^ and small/medium at 18, i.e. lean during adolescence and adulthood
			HR (95% CI)*	HR (95% CI)†		HR (95% CI)‡		HR (95% CI)§
Depression	Small	5266	1.00 (Reference)	1.00 (Reference)	2973	1.00 (Reference)	2905	1.00 (Reference)
	Medium	3369	**0.95 (0.91 to 0.99)**	**0.90 (0.86 to 0.94)**	1483	**0.92 (0.87 to 0.98)**	1345	**0.92 (0.86 to 0.98)**
	Large	1007	1.05 (0.98 to 1.12)	0.95 (0.89 to 1.02)	387	1.03 (0.92 to 1.14)	263	1.02 (0.90 to 1.15)
	Trend	9642	1.00 (0.97 to 1.03)	**0.95 (0.92 to 0.98)**	4843	0.97 (0.93 to 1.02)	4513	0.96 (0.92 to 1.01)
	Continuous	9642	1.00 (0.99 to 1.02)	**0.97 (0.96 to 0.99)**	4843	0.99 (0.96 to 1.01)	4513	0.98 (0.96 to 1.01)
Anorexia	Small	194	1.00 (Reference)	1.00 (Reference)	156	1.00 (Reference)	150	1.00 (Reference)
	Medium	228	**1.74 (1.43 to 2.10)**	**2.22 (1.83 to 2.69)**	196	**2.36 (1.91 to 2.91)**	180	**2.41 (1.94 to 3.00)**
	Large	76	**2.13 (1.63 to 2.77)**	**3.18 (2.44 to 4.16)**	57	**2.94 (2.17 to 3.99)**	38	**2.89 (2.02 to 4.13)**
	Trend	498	**1.51 (1.34 to 1.71)**	**1.86 (1.65 to 2.10)**	409	**1.84 (1.61 to 2.10)**	368	**1.89 (1.64 to 2.19)**
	Continuous	498	**1.25 (1.18 to 1.33)**	**1.40 (1.32 to 1.49)**	409	**1.39 (1.30 to 1.48)**	368	**1.43 (1.33 to 1.54)**
Bulimia	Small	121	1.00 (Reference)	1.00 (Reference)	89	1.00 (Reference)	79	1.00 (Reference)
	Medium	154	**1.85 (1.46 to 2.34)**	**1.92 (1.51 to 2.44)**	101	**2.11 (1.58 to 2.80)**	84	**2.13 (1.57 to 2.89)**
	Large	43	**1.91 (1.35 to 2.70)**	**2.04 (1.43 to 2.91)**	21	**1.92 (1.19 to 3.09)**	16	**2.36 (1.38 to 4.04)**
	Trend	318	**1.48 (1.27 to 1.72)**	**1.53 (1.31 to 1.79)**	211	**1.58 (1.30 to 1.91)**	179	**1.72 (1.39 to 2.13)**
	Continuous	318	**1.24 (1.15 to 1.34)**	**1.27 (1.17 to 1.37)**	211	**1.31 (1.19 to 1.44)**	179	**1.37 (1.24 to 1.53)**
PCOS	Small	102	1.00 (Reference)	1.00 (Reference)	59	1.00 (Reference)	57	1.00 (Reference)
	Medium	83	1.16 (0.87 to 1.55)	1.00 (0.74 to 1.34)	33	1.03 (0.67 to 1.58)	27	0.94 (0.60 to 1.49)
	Large	32	**1.69 (1.13 to 2.51)**	1.25 (0.83 to 1.88)	12	1.71 (0.92 to 3.18)	10	2.11 (1.08 to 4.13)
	Trend	217	**1.26 (1.04 to 1.53)**	1.08 (0.89 to 1.32)	104	1.21 (0.90 to 1.61)	94	1.23 (0.89 to 1.70)
	Continuous	217	**1.14 (1.04 to 1.25)**	1.05 (0.95 to 1.16)	104	1.08 (0.93 to 1.26)	94	1.09 (0.92 to 1.29)
Ovarian cyst	Small	2376	1.00 (Reference)	1.00 (Reference)	1374	1.00 (Reference)	1347	1.00 (Reference)
	Medium	1501	0.95 (0.89 to 1.01)	0.93 (0.87 to 0.99)	727	0.98 (0.90 to 1.08)	663	0.98 (0.90 to 1.08)
	Large	448	1.04 (0.94 to 1.15)	1.00 (0.91 to 1.11)	189	1.07 (0.92 to 1.25)	141	1.17 (0.98 to 1.39)
	Trend	4325	0.99 (0.95 to 1.04)	0.98 (0.93 to 1.02)	2290	1.01 (0.95 to 1.08)	2151	1.03 (0.96 to 1.11)
	Continuous	4325	1.00 (0.98 to 1.02)	0.99 (0.97 to 1.01)	2290	1.00 (0.97 to 1.04)	2151	1.01 (0.97 to 1.04)
Diabetes	Small	958	1.00 (Reference)	1.00 (Reference)	296	1.00 (Reference)	291	1.00 (Reference)
	Medium	623	1.01 (0.91 to 1.11)	**0.85 (0.77 to 0.94)**	140	0.88 (0.72 to 1.07)	128	0.88 (0.71 to 1.08)
	Large	224	**1.34 (1.16 to 1.55)**	0.97 (0.84 to 1.13)	36	0.92 (0.65 to 1.31)	25	0.94 (0.62 to 1.41)
	Trend	1805	**1.11 (1.04 to 1.19)**	0.94 (0.88 to 1.01)	472	0.93 (0.80 to 1.07)	444	0.92 (0.78 to 1.08)
	Continuous	1805	**1.04 (1.01 to 1.08)**	**0.95 (0.92 to 0.98)**	472	0.95 (0.88 to 1.02)	444	0.95 (0.88 to 1.03)
Hyperlipidemia	Small	3864	1.00 (Reference)	1.00 (Reference)	1829	1.00 (Reference)	1797	1.00 (Reference)
	Medium	2286	**0.92 (0.87 to 0.97)**	**0.86 (0.82 to 0.91)**	824	**0.82 (0.76 to 0.89)**	760	**0.83 (0.76 to 0.90)**
	Large	719	1.08 (0.99 to 1.17)	0.96 (0.89 to 1.04)	241	0.98 (0.86 to 1.12)	175	1.05 (0.90 to 1.22)
	Trend	6869	0.99 (0.96 to 1.03)	**0.94 (0.90 to 0.97)**	2894	**0.92 (0.87 to 0.97)**	2732	**0.92 (0.87 to 0.98)**
	Continuous	6869	0.99 (0.98 to 1.01)	**0.96 (0.95 to 0.98)**	2894	**0.95 (0.92 to 0.98)**	2732	**0.95 (0.92 to 0.98)**
Stroke	Small	422	1.00 (Reference)	1.00 (Reference)	189	1.00 (Reference)	186	1.00 (Reference)
	Medium	202	**0.75 (0.64 to 0.89)**	**0.71 (0.60 to 0.84)**	93	0.92 (0.72 to 1.18)	85	0.92 (0.71 to 1.18)
	Large	65	0.90 (0.70 to 1.17)	0.81 (0.62 to 1.06)	22	0.88 (0.56 to 1.36)	13	0.75 (0.43 to 1.32)
	Trend	689	**0.87 (0.77 to 0.98)**	**0.82 (0.73 to 0.93)**	304	0.93 (0.78 to 1.11)	284	0.89 (0.73 to 1.09)
	Continuous	689	**0.94 (0.88 to 0.99)**	**0.91 (0.86 to 0.96)**	304	0.96 (0.88 to 1.05)	284	0.94 (0.85 to 1.04)
Heart failure	Small	204	1.00 (Reference)	1.00 (Reference)	81	1.00 (Reference)	74	1.00 (Reference)
	Medium	130	1.02 (0.82 to 1.27)	0.91 (0.73 to 1.14)	43	0.99 (0.68 to 1.43)	39	1.05 (0.72 to 1.55)
	Large	34	1.00 (0.70 to 1.44)	0.82 (0.57 to 1.18)	7	0.64 (0.30 to 1.39)	4	0.58 (0.21 to 1.59)
	Trend	368	1.01 (0.86 to 1.18)	0.91 (0.78 to 1.06)	131	0.89 (0.67 to 1.17)	117	0.92 (0.68 to 1.25)
	Continuous	368	1.01 (0.94 to 1.09)	0.96 (0.89 to 1.04)	131	0.95 (0.83 to 1.09)	117	0.97 (0.83 to 1.13)
Myocardial infarction	Small	277	1.00 (Reference)	1.00 (Reference)	103	1.00 (Reference)	103	1.00 (Reference)
	Medium	146	0.83 (0.68 to 1.02)	0.76 (0.62 to 0.93)	50	0.90 (0.64 to 1.26)	43	0.83 (0.58 to 1.18)
	Large	56	1.20 (0.90 to 1.60)	1.00 (0.74 to 1.33)	14	1.00 (0.57 to 1.75)	9	0.93 (0.47 to 1.83)
	Trend	479	1.00 (0.87 to 1.15)	0.91 (0.79 to 1.04)	167	0.96 (0.76 to 1.22)	155	0.89 (0.68 to 1.17)
	Continuous	479	0.99 (0.93 to 1.06)	0.94 (0.88 to 1.01)	167	0.95 (0.84 to 1.07)	155	0.91 (0.79 to 1.04)
Angina pectoris	Small	339	1.00 (Reference)	1.00 (Reference)	128	1.00 (Reference)	127	1.00 (Reference)
	Medium	180	0.84 (0.70 to 1.01)	**0.77 (0.64 to 0.92)**	58	0.84 (0.62 to 1.15)	51	0.80 (0.58 to 1.10)
	Large	51	0.89 (0.66 to 1.19)	0.75 (0.55 to 1.00)	7	**0.40 (0.19 to 0.86)**	3	**0.25 (0.08 to 0.78)**
	Trend	570	0.90 (0.79 to 1.02)	**0.82 (0.72 to 0.94)**	193	**0.74 (0.58 to 0.95)**	181	**0.69 (0.53 to 0.91)**
	Continuous	570	0.94 (0.89 to 1.00)	**0.90 (0.84 to 0.96)**	193	**0.85 (0.76 to 0.96)**	181	**0.82 (0.72 to 0.94)**

Statistically significant associations (P < 0.05) are presented in bold. ^*^Stratified by year of birth (1950, 1951–1960, 1961 and later). ^†^Stratified by year of birth and adjusted for body mass index (BMI, kg/m^2^, continuous) at questionnaire. ^‡^Subset of women with adult BMI less than 25 kg/m^2^, stratified by year of birth. ^**§**^Subset of women with adult BMI less than 25 kg/m and either small or medium body size at age 18, stratified by year of birth.

† = 0.331, respectively). No clear dose-dependent associations were observed for depression, ovarian cyst, stroke, hyperlipidemia, heart failure, myocardial infarction and angina pectoris.

**Figure 2:**
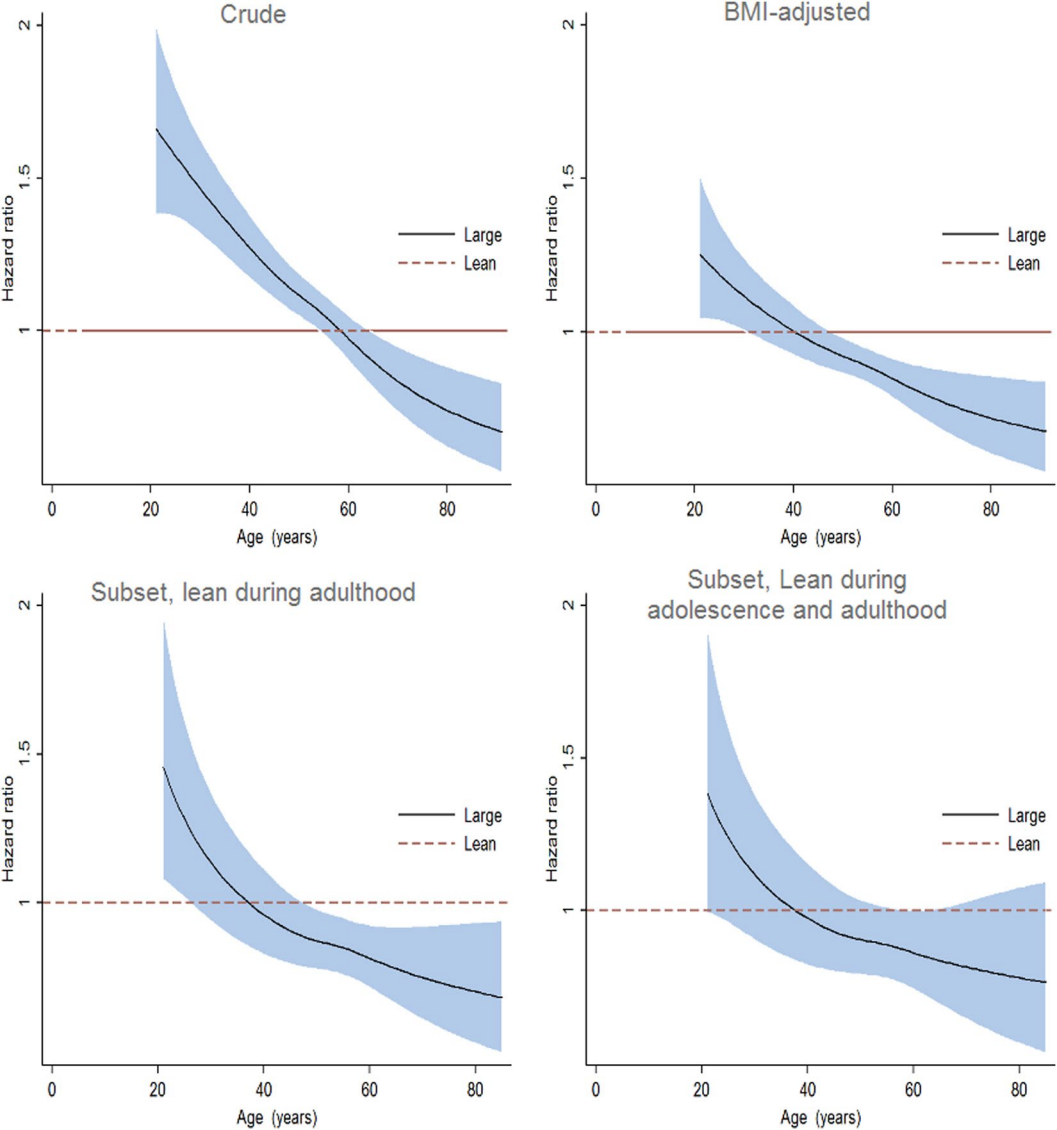
Age-dependent hazard ratios (HRs) and corresponding 95% confidence intervals (CIs) for hypertension, large versus small childhood body size. HRs were estimated from flexible parametric survival models adjusting for year of birth (crude); adjusting for year of birth and BMI (BMI-adjusted); restricted to a subset of women with adult BMI less than 25 kg/m^2^ and adjusting for year of birth (lean during adulthood); and restricted to subset of women with adult BMI less than 25 kg/m^2^ and either small or medium body size at age 18 (lean during adolescence and adulthood).

The HR and corresponding 95% CI changed over time for the association between childhood body size and hypertension (proportional hazards assumption violated for this outcome), and was thus examined using flexible parametric models (Fig. 2). In the crude analyses, childhood body size was associated with increased risk of hypertension before age 60, but not afterwards. The increased risk was attenuated when adjusted for adult BMI. Similarly, larger childhood body size did not appreciably elevate adult hypertension risk among women who were lean during adolescence or adulthood.

In the sensitivity analyses, we did not find any appreciable changes to the results associated with non-breast cancer outcomes when restricting to non-breast cancer participants (Supplementary Tables 2 and 3).

## Discussion

In summary, our results suggest that childhood body size is an important risk factor for breast cancer and eating disorders that is independent of adult BMI. The associations between childhood body size with other adult diseases such as diabetes, PCOS and hypertension have to be considered in the light of adult BMI.

An inverse relationship between childhood body size and breast cancer has been consistently shown^1^. As adult obesity is an established risk factor for postmenopausal but not premenopausal breast cancer, the apparent protective effect that a large childhood body size has over a small childhood body size for both pre- and postmenopausal breast cancer has been much discussed^40–43^. Considering that childhood fatness is typically associated with earlier menarche, which is in turn associated with high breast cancer risk, the inverse correlation between childhood body size and breast cancer risk is counterintuitive. However, the inverse association has been shown not to be mediated by adult BMI or menstrual characteristics^44,45^, which suggests that an excess of adiposity during childhood might prevent tumorigenesis through other mechanisms. A possible explanation is that the increased exposure to estrogen from the extra adipose tissue at a young age induces a persistent upregulation of tumor suppressor genes^46^.

While much interest and attention is generated by the conclusion that a large childhood body size reduces breast cancer risk, it is important to evaluate childhood body size alongside other health outcomes. Not unexpectedly, obesity in childhood has been shown to be a retrospective correlate of eating disorders such as bulimia in later years^47^. In a large UK-based study, Micali *et al*. identified body dissatisfaction in children as young as eight years old to be prospectively associated with dieting and purging behaviors during adolescence^48^. Discontent with the size and shape of their body might subject children with larger body size to undue pressure from media, family and peers, putting them at risk of eating disorders in their teens.

Other common diseases that are hypothesized to have beginnings in early life include a range of cancers (other than breast cancer) and several adult obesity-related morbidities^3^. A high BMI in childhood has been shown to frequently persist into adult life, where obese children were five-fold more likely to remain obese later on in life compared to those who were not obese^49,50^. As our data showed that the increased risks of diabetes and hypertension conferred by larger childhood body size do not persist after controlling for adult BMI, it is likely that the associations between childhood body size and these diseases are driven by adult BMI. Similarly, in the Nurses’ Health Study, it was observed that children who were of large body size were not associated with an increased risk of diabetes if they became lean in later years^51^.

Unexpectedly, no clear link between childhood body size and cardiovascular diseases was found in our study. However, a systematic review and meta-analysis of more than 30 studies by Llewellyn *et al*. suggested that childhood BMI is of limited accuracy in predicting adult morbidity, reason being that less than a third of the adult morbidities developed in children who were overweight or obese^3^. On the contrary, the majority of adult chronic diseases (~70%) occurred in children who were of normal weight^3,49^, which could possibly explain the absence of associations between childhood body size and cardiovascular diseases in adulthood. Other studies have also reported that an independent contribution of childhood body size in affecting blood lipid status, insulin levels, metabolic syndrome or cardiovascular disease is unlikely^52,53^.

The main strength of this study lies in its large sample size of close to 70,000 women. Other works that have simultaneously examined multiple long-term health risk outcomes with respect to childhood anthropometry in a single study include smaller studies such as the Scottish Mental Survey of 1947 (4,620 participants, of which approximately half are female), a Swedish prospective study of 504 children (271 girls) with 40 years of follow-up and the Harvard Growth Study of 1922 to 1935 (508 lean or overweight adolescents aged between 13 to 18)^54–56^. An additional strength of this study is that cancer outcomes were identified through the long-standing nation-wide Swedish Cancer Registry, to which the reporting of all newly diagnosed cancer cases is compulsory. The Swedish Cancer Registry was founded in 1958 and shows an almost complete coverage due to extensive monitoring and requests for missing data^36^.

While the five cancer outcomes were retrieved from Swedish registries, it should be noted that the study used self-reported medical histories of the participants to study seven other common diseases, for example, hypertension and diabetes, which are typically diagnosed in primary health care^57^. Unlike inpatient or hospital discharge records (complete coverage since 1987^58^) or hospital-based outpatient physician visits (from 2011) which can be retrieved from the Swedish National Patient Register, primary health care data is not tracked on a national level. However, good agreement has been shown between patient-reported comorbidity events and information abstracted from medical records^59^. As with all studies using survey-based data regarding events or experiences from the past, recall error due to time and memory failure is a limitation and can bias our estimates towards the null. This is especially true for the conditions which usually affect younger women, such as polycystic ovary syndrome, ovarian cysts, depression, bulimia and anorexia. However, individuals are unlikely to forget if they have ever been diagnosed with one of these conditions. It is also possible that women participating in a mammography screening program may have a differential recall of non-breast related health events. Another point to note is that since KARMA comprises participants of a nation-wide mammography screening program, the generalizability of our results may be limited to a more health-conscious group of women. However, since the Swedish health care system is mainly government-funded and decentralized and all women essentially have the same access, the study should represent women from a wide range of income levels and backgrounds. As childhood BMI was not collected, it was not possible to validate how closely recalled somatotypes aligned with childhood BMI z-score. However, this measure is well-validated by others^32,33^ and is currently used to assess recalled somatotypes in several large, ongoing prospective studies, including the Nurses’ Health Study^60^.

Our findings provide perspective for the wide range of long-term consequences of childhood body size on later life well-being. While further research on inverse association between childhood body size and breast cancer will help us to better understand the etiology of the disease, other negative health outcomes of childhood obesity in adult life need to be weighed simultaneously in the light of the apparent protective effect against breast cancer.

### Availability of data and Materials

The datasets used and/or analysed during the current study are available from the corresponding author on reasonable request.

### Ethical approval and consent to participate

Each participant gave informed consent and this study has been approved by the ethical review board at Karolinska Institutet. All analyses were performed in accordance with relevant institutional and national guidelines and regulations.

## Electronic supplementary material

Supplementary Tables 1–3
