# Supplementary material for: Associations between childhood body size and seventeen adverse outcomes: analysis of 65,057 European women
Source: Sci Rep. 2017 Dec 5;7:16917. doi: 10.1038/s41598-017-17258-5 (PMC5717076; doi:10.1038/s41598-017-17258-5)
Supplement: Supplementary file 1 — Supplementary Tables 1–3 [file 41598_2017_17258_MOESM1_ESM.pdf]

Associations between childhood body size and seventeen adverse outcomes: analysis of 65,057 European women

Jingmei Li, assistant professor[1,2,3]*, Mikael Eriksson, doctoral candidate[3], Wei He, postdoctoral researcher[3], Per Hall, professor[3,4], Kamila Czene, professor[3]

[1] Genome Institute of Singapore, 60 Biopolis Street, Genome, #02-01, Singapore 138672, Singapore

[2] Department Of Surgery, Yong Loo Lin School of Medicine, National University of Singapore, Singapore

[3] Karolinska Institutet, Department of Medical Epidemiology and Biostatistics, Box 281, 171 77 Stockholm, Sweden

[4] Department of Oncology, Södersjukhuset, 118 84 Stockholm, Sweden

*Correspondence to:

Jingmei Li, Genome Institute of Singapore, 60 Biopolis Street, Genome, #02-01, Singapore 138672, Singapore
Tel: +65 6808 8312 Email: lijm1@gis.a-star.edu.sg

Keywords: childhood body size, somatotype, breast cancer, hypertension, diabetes

Supplementary Table 1 Overlap between cases with breast cancer and cases with other negative health outcomes.

	**Overlap, *n* (%)**
Cancer outcomes	
Cervical cancer	9 (4.1)
Uterine	21 (8.1)
Melanoma	22 (4.0)
Colon	17 (7.2)
Non-cancer outcomes	
Depression	360 (3.7)
Anorexia	10 (2.0)
Bulimia	8 (2.5)
PCOS	4 (1.8)
Ovarian cyst	132 (3.1)
Stroke	46 (6.7)
Diabetes	102 (5.7)
Hypertension	693 (5.5)
Hyperlipidemia	377 (5.5)
Heart failure	31 (8.4)
Myocardial infarction	46 (9.6)
Angina pectoris	35 (6.1)

Supplementary Table 2 Hazard ratios (HR) and corresponding 95% confidence intervals (CI) for the associations between childhood body size and different cancers in 62,324 breast cancer controls. Statistically significant associations (P<0.05) are presented in bold.

			Crude	Adjusted for BMI		Subset, adult BMI<25kg/m², i.e. lean during adulthood		Subset, adult BMI<25kg/m² and small/medium at 18, i.e. lean during adolescence and adulthood
**Cancer outcome**	**Body size**	***n***	**HR (95% CI)***	**HR (95% CI)†**	***n*‡**	**HR (95% CI)‡**	***n*§**	**HR (95% CI)§**
Cervical	Small	125	1.00 (Reference)	1.00 (Reference)	75	1.00 (Reference)	75	1.00 (Reference)
	Medium	66	0.79 (0.59 to 1.07)	0.79 (0.59 to 1.07)	30	0.74 (0.49 to 1.13)	29	0.77 (0.50 to 1.18)
	Large	22	0.96 (0.61 to 1.52)	0.96 (0.61 to 1.52)	14	1.42 (0.80 to 2.52)	8	1.15 (0.56 to 2.39)
	Trend	213	0.91 (0.74 to 1.12)	0.91 (0.74 to 1.12)	119	1.02 (0.77 to 1.35)	112	0.92 (0.67 to 1.26)
	Continuous	213	0.95 (0.86 to 1.05)	0.95 (0.86 to 1.05)	119	0.98 (0.85 to 1.13)	112	0.92 (0.79 to 1.08)
Uterine	Small	141	1.00 (Reference)	1.00 (Reference)	66	1.00 (Reference)	63	1.00 (Reference)
	Medium	78	0.87 (0.66 to 1.15)	0.81 (0.61 to 1.07)	26	0.73 (0.46 to 1.15)	19	0.60 (0.36 to 1.00)
	Large	19	0.80 (0.50 to 1.29)	0.70 (0.43 to 1.14)	6	0.66 (0.28 to 1.51)	3	0.49 (0.15 to 1.56)
	Trend	238	0.89 (0.73 to 1.08)	0.83 (0.68 to 1.01)	98	0.77 (0.55 to 1.08)	85	0.64 (0.42 to 0.96)
	Continuous	238	0.97 (0.88 to 1.07)	0.94 (0.85 to 1.03)	98	0.92 (0.79 to 1.08)	85	0.85 (0.70 to 1.02)
Melanoma	Small	287	1.00 (Reference)	1.00 (Reference)	176	1.00 (Reference)	172	1.00 (Reference)
	Medium	178	0.95 (0.79 to 1.14)	0.95 (0.79 to 1.15)	90	0.95 (0.74 to 1.22)	81	0.94 (0.72 to 1.22)
	Large	64	1.25 (0.95 to 1.64)	1.26 (0.96 to 1.66)	23	0.99 (0.64 to 1.52)	17	1.06 (0.64 to 1.75)
	Trend	529	1.06 (0.94 to 1.20)	1.07 (0.94 to 1.21)	289	0.97 (0.81 to 1.17)	270	0.99 (0.81 to 1.20)
	Continuous	529	1.02 (0.96 to 1.09)	1.03 (0.96 to 1.09)	289	0.98 (0.90 to 1.08)	270	0.99 (0.90 to 1.10)
Colon	Small	119	1.00 (Reference)	1.00 (Reference)	65	1.00 (Reference)	64	1.00 (Reference)
	Medium	76	1.00 (0.75 to 1.33)	1.00 (0.75 to 1.34)	36	1.03 (0.69 to 1.55)	32	1.00 (0.66 to 1.53)
	Large	23	1.13 (0.72 to 1.76)	1.13 (0.72 to 1.77)	13	1.49 (0.82 to 2.70)	8	1.34 (0.64 to 2.79)
	Trend	218	1.04 (0.85 to 1.27)	1.04 (0.85 to 1.27)	114	1.15 (0.88 to 1.52)	104	1.09 (0.80 to 1.48)
	Continuous	218	1.02 (0.93 to 1.13)	1.02 (0.93 to 1.13)	114	1.09 (0.95 to 1.25)	104	1.08 (0.92 to 1.25)

* Stratified by year of birth (1950, 1951-1960, 1961 and later).

† Stratified by year of birth and adjusted for body mass index (BMI, kg/m², continuous) at questionnaire.

‡ Subset of women with adult BMI less than 25 kg/m², stratified by year of birth.

§ Subset of women with adult BMI less than 25 kg/m and either small or medium body size at age 18, stratified by year of birth.

Supplementary Table 3 Hazard ratios (HR) and corresponding 95% confidence intervals (CI) for the associations between childhood body size and self-reported common diseases in 62,324 breast cancer controls. Statistically significant associations (P<0.05) are presented in bold.

			**Crude**	**Adjusted for BMI**		**Subset, adult BMI<25kg/m^2, i.e. lean during adulthood**		**Subset, adult BMI<25kg/m^2 and small/medium at 18, i.e. lean during adolescence and adulthood**
**Outcome**	**Body size**	***n***	**HR (95% CI)***	**HR (95% CI)†**	***n*‡**	**HR (95% CI)‡**	***n*§**	**HR (95% CI)§**
Depression	Small	5080	1.00 (Reference)	1.00 (Reference)	2876	1.00 (Reference)	2808	1.00 (Reference)
	Medium	3236	**0.94 (0.90 to 0.98)**	**0.89 (0.85 to 0.93)**	1426	**0.91 (0.86 to 0.97)**	1292	**0.91 (0.85 to 0.97)**
	Large	966	1.03 (0.96 to 1.11)	0.93 (0.87 to 1.00)	371	1.01 (0.90 to 1.12)	254	1.00 (0.88 to 1.14)
	Trend	9282	0.99 (0.96 to 1.02)	**0.94 (0.91 to 0.97)**	4673	0.96 (0.92 to 1.01)	4354	0.96 (0.91 to 1.00)
	Continuous	9282	1.00 (0.98 to 1.01)	0.97 (0.96 to 0.99)	4673	0.98 (0.96 to 1.00)	4354	0.98 (0.95 to 1.00)
Anorexia	Small	191	1.00 (Reference)	1.00 (Reference)	153	1.00 (Reference)	147	1.00 (Reference)
	Medium	223	**1.72 (1.42 to 2.09)**	**2.19 (1.80 to 2.66)**	191	**2.34 (1.89 to 2.89)**	176	**2.40 (1.93 to 2.99)**
	Large	74	**2.08 (1.59 to 2.72)**	**3.11 (2.37 to 4.08)**	55	**2.87 (2.11 to 3.90)**	37	**2.83 (1.97 to 4.06)**
	Trend	488	**1.50 (1.32 to 1.69)**	**1.84 (1.63 to 2.08)**	399	**1.82 (1.59 to 2.08)**	360	**1.88 (1.62 to 2.18)**
	Continuous	488	**1.24 (1.17 to 1.32)**	**1.39 (1.31 to 1.48)**	399	**1.38 (1.29 to 1.48)**	360	**1.43 (1.33 to 1.53)**
Bulimia	Small	119	1.00 (Reference)	1.00 (Reference)	87	1.00 (Reference)	77	1.00 (Reference)
	Medium	149	**1.81 (1.42 to 2.31)**	**1.90 (1.49 to 2.42)**	98	**2.09 (1.56 to 2.78)**	81	**2.10 (1.54 to 2.87)**
	Large	42	**1.88 (1.32 to 2.67)**	**2.04 (1.42 to 2.91)**	21	**1.95 (1.21 to 3.14)**	16	**2.39 (1.40 to 4.10)**
	Trend	310	**1.46 (1.25 to 1.71)**	**1.52 (1.30 to 1.78)**	206	**1.58 (1.30 to 1.91)**	174	**1.72 (1.39 to 2.14)**
	Continuous	310	**1.24 (1.15 to 1.33)**	**1.27 (1.17 to 1.37)**	206	**1.31 (1.19 to 1.44)**	174	**1.38 (1.24 to 1.53)**
PCOS	Small	100	1.00 (Reference)	1.00 (Reference)	59	1.00 (Reference)	57	1.00 (Reference)
	Medium	81	1.15 (0.86 to 1.55)	0.99 (0.74 to 1.34)	33	1.03 (0.67 to 1.58)	27	0.94 (0.60 to 1.49)
	Large	32	**1.71 (1.15 to 2.55)**	1.27 (0.84 to 1.92)	12	1.70 (0.91 to 3.16)	10	**2.09 (1.07 to 4.09)**
	Trend	213	**1.27 (1.05 to 1.54)**	1.09 (0.90 to 1.33)	104	1.21 (0.90 to 1.61)	94	1.23 (0.89 to 1.70)
	Continuous	213	**1.14 (1.03 to 1.25)**	1.05 (0.95 to 1.16)	104	1.08 (0.93 to 1.26)	94	1.09 (0.92 to 1.28)
Ovarian cyst	Small	2303	1.00 (Reference)	1.00 (Reference)	1335	1.00 (Reference)	1308	1.00 (Reference)
	Medium	1450	0.94 (0.88 to 1.00)	**0.92 (0.86 to 0.99)**	703	0.97 (0.89 to 1.07)	641	0.98 (0.89 to 1.07)
	Large	440	1.04 (0.94 to 1.15)	1.00 (0.91 to 1.11)	186	1.07 (0.92 to 1.25)	139	1.17 (0.98 to 1.40)
	Trend	4193	0.99 (0.95 to 1.04)	0.97 (0.93 to 1.02)	2224	1.01 (0.95 to 1.08)	2088	1.03 (0.96 to 1.11)
	Continuous	4193	1.00 (0.98 to 1.02)	0.99 (0.97 to 1.01)	2224	1.00 (0.97 to 1.04)	2088	1.01 (0.97 to 1.04)
Diabetes	Small	899	1.00 (Reference)	1.00 (Reference)	279	1.00 (Reference)	274	1.00 (Reference)
	Medium	590	1.01 (0.91 to 1.12)	0.85 (0.77 to 0.95)	135	0.89 (0.73 to 1.10)	124	0.90 (0.73 to 1.11)
	Large	214	**1.35 (1.16 to 1.57)**	0.98 (0.84 to 1.14)	34	0.91 (0.64 to 1.30)	24	0.93 (0.62 to 1.42)
	Trend	1703	**1.11 (1.04 to 1.19)**	0.95 (0.88 to 1.02)	448	0.93 (0.80 to 1.08)	422	0.93 (0.79 to 1.10)
	Continuous	1703	**1.04 (1.01 to 1.08)**	**0.95 (0.92 to 0.99)**	448	0.95 (0.88 to 1.02)	422	0.95 (0.88 to 1.03)
Hyperlipidemia	Small	3647	1.00 (Reference)	1.00 (Reference)	1728	1.00 (Reference)	1698	1.00 (Reference)
	Medium	2161	**0.91 (0.86 to 0.96)**	**0.85 (0.81 to 0.90)**	773	**0.81 (0.75 to 0.89)**	712	**0.82 (0.75 to 0.89)**
	Large	684	1.07 (0.98 to 1.16)	0.95 (0.88 to 1.03)	230	0.97 (0.85 to 1.12)	167	1.03 (0.88 to 1.21)
	Trend	6492	0.99 (0.95 to 1.03)	**0.93 (0.90 to 0.97)**	2731	**0.91 (0.86 to 0.97)**	2577	**0.92 (0.86 to 0.98)**
	Continuous	6492	0.99 (0.97 to 1.01)	**0.96 (0.94 to 0.98)**	2731	**0.95 (0.92 to 0.98)**	2577	**0.95 (0.92 to 0.98)**
Stroke	Small	391	1.00 (Reference)	1.00 (Reference)	178	1.00 (Reference)	175	1.00 (Reference)
	Medium	191	**0.76 (0.64 to 0.90)**	**0.72 (0.60 to 0.85)**	85	0.89 (0.68 to 1.15)	78	0.89 (0.68 to 1.16)
	Large	61	0.90 (0.69 to 1.18)	0.81 (0.62 to 1.06)	22	0.92 (0.59 to 1.43)	13	0.78 (0.45 to 1.38)
	Trend	643	**0.87 (0.77 to 0.98)**	**0.82 (0.73 to 0.93)**	285	0.93 (0.77 to 1.12)	266	0.89 (0.72 to 1.09)
	Continuous	643	**0.94 (0.88 to 0.99)**	**0.91 (0.86 to 0.97)**	285	0.95 (0.87 to 1.04)	266	0.93 (0.84 to 1.03)
Heart failure	Small	188	1.00 (Reference)	1.00 (Reference)	78	1.00 (Reference)	71	1.00 (Reference)
	Medium	119	1.00 (0.79 to 1.26)	0.90 (0.71 to 1.13)	39	0.93 (0.63 to 1.37)	36	1.01 (0.68 to 1.51)
	Large	30	0.94 (0.64 to 1.39)	0.78 (0.53 to 1.15)	5	0.47 (0.19 to 1.16)	3	0.44 (0.14 to 1.41)
	Trend	337	0.98 (0.83 to 1.15)	0.89 (0.75 to 1.05)	122	0.81 (0.60 to 1.09)	110	0.86 (0.62 to 1.19)
	Continuous	337	1.01 (0.93 to 1.09)	0.95 (0.88 to 1.03)	122	0.92 (0.79 to 1.06)	110	0.94 (0.80 to 1.10)
Myocardial infarction	Small	252	1.00 (Reference)	1.00 (Reference)	93	1.00 (Reference)	93	1.00 (Reference)
	Medium	131	0.81 (0.65 to 1.00)	**0.73 (0.59 to 0.90)**	46	0.91 (0.64 to 1.30)	39	0.83 (0.57 to 1.21)
	Large	50	1.16 (0.85 to 1.57)	0.96 (0.70 to 1.30)	13	1.01 (0.57 to 1.81)	8	0.89 (0.43 to 1.83)
	Trend	433	0.98 (0.85 to 1.13)	0.89 (0.77 to 1.03)	152	0.97 (0.76 to 1.24)	140	0.89 (0.67 to 1.18)

	Continuous	433	0.98 (0.91 to 1.05)	0.93 (0.87 to 1.00)	152	0.96 (0.85 to 1.09)	140	0.91 (0.79 to 1.05)
Angina pectoris	Small	318	1.00 (Reference)	1.00 (Reference)	121	1.00 (Reference)	120	1.00 (Reference)
	Medium	170	0.84 (0.70 to 1.01)	**0.76 (0.63 to 0.92)**	53	0.81 (0.59 to 1.12)	46	0.76 (0.54 to 1.07)
	Large	47	0.86 (0.63 to 1.17)	**0.72 (0.53 to 0.98)**	7	**0.42 (0.20 to 0.90)**	3	**0.26 (0.08 to 0.81)**
	Trend	535	0.89 (0.78 to 1.02)	**0.81 (0.71 to 0.93)**	181	**0.73 (0.57 to 0.94)**	169	**0.67 (0.50 to 0.89)**
	Continuous	535	0.94 (0.88 to 1.00)	**0.90 (0.84 to 0.96)**	181	**0.85 (0.75 to 0.96)**	169	**0.81 (0.71 to 0.93)**

* Stratified by year of birth (1950, 1951-1960, 1961 and later).

† Stratified by year of birth and adjusted for body mass index (BMI, kg/m^2, continuous) at questionnaire.

‡ Subset of women with adult BMI less than 25 kg/m^2, stratified by year of birth.

§ Subset of women with adult BMI less than 25 kg/m and either small or medium body size at age 18, stratified by year of birth.
